# A Comprehensive Study on Ruptured Liver Abscess in a Tertiary Care Center

**DOI:** 10.7759/cureus.64526

**Published:** 2024-07-14

**Authors:** Arun Singh, Shivani B Paruthy, Vaibhav Kuraria, Mohit Dhawaria, Dhananjay Khera, Hrishikesh M S, Hinduja Raju, Singamsetty S Madhuri, Yogesh Saini, Abhinav Kumar

**Affiliations:** 1 General Surgery, Vardhman Mahavir Medical College and Safdarjung Hospital, New Delhi, IND

**Keywords:** liver abscess, percutaneus catheter drainage, extended right hemicolectomy, pigtail catheter drainage, ruptured liver abscess, emergency exploratory laparotomy, liver abscess drainage

## Abstract

Background

Bacteria and parasites cause liver abscesses (LAs), with the unusual but fatal consequence of ruptured LA. Along with the clinical signs of icterus, right upper quadrant pain, and a history of loose stools, patients present with non-specific symptoms such as fever, nausea, and generalized weakness. Consistent findings include male sex prevalence and frequent alcohol consumption. Leukocytosis, abnormal liver function, and an increased international normalized ratio have been identified by biochemical analysis; however, these findings are not specific to a ruptured LA diagnosis, and imaging is necessary to reach a definitive diagnosis. Ultrasonography usually confirms the diagnosis, and computed tomography is required in certain situations. In confined ruptures, percutaneous drainage combined with antibiotic therapy is typically the initial treatment course. Generally reserved for non-responders or moribund patients with delayed presentation, an open surgical approach may involve simple draining of a ruptured abscess or ileocecal resection, or right hemicolectomy in cases of large bowel perforations, both of which increase patient morbidity. A definite guide to management is still missing in the literature. In this article, we have discussed and correlated with data the predictors of surgery and preoperative predictors of perforation.

Materials and methods

This retrospective study was performed at Safdarjung Hospital, New Delhi, between January 2022 and December 2023. The study included 115 patients diagnosed with ruptured LA by ultrasound. Medical records were analyzed, and various parameters of the illness, clinical features, hematological and biochemical profiles, ultrasound features, and therapeutic measures were noted and assessed.

Results

Of the 115 patients, 88% (n = 101) were male. The most common symptoms were abdominal pain (114 patients) and right upper abdominal tenderness (107 patients). Fifty-two patients were treated with percutaneous drainage, and 42 underwent laparotomy. Intercostal drainage (ICD) tubes were placed in 19 patients. Sixteen patients had large bowel perforations. Twenty-three patients died (20%), including 17 patients who underwent laparotomy and nine patients who had large bowel perforation (39.1% associated with overall mortality, 52.9% associated with mortality in laparotomy). One patient with percutaneous drainage and a right ICD tube died in the intensive care unit. Four patients died before intervention. Significant associations were noted between perforation and mortality in patients who underwent surgical drainage. Loose motions, alcohol and smoking consumption, and deranged creatinine and albumin levels were found to have a significant association with surgical drainage.

Conclusion

The study found that a ruptured liver abscess (LA) may require surgery to drain the collection or repair the pathological bowel, which increases the morbidity, but it is a lifesaving procedure over percutaneous catheter drainage. The study also identified factors associated with a higher risk of death, such as a history of loose stools and low blood albumin levels.

## Introduction

The first recorded cases of liver abscess (LA) date back to 400 B.C. in ancient Greece, when Hippocrates believed that the sort of fluid in the lesion would determine patient outcome. One potentially fatal consequence of bacterial, fungal, protozoan, and worm infections is LA, a confined, frequently encysted, purulent inflammation with parenchymal necrosis [1]. LAs have a mortality rate of up to 20% and are classified into different categories based on their origin, and the two most common varieties are pyogenic LA (PLA) and amebic LA (ALA) [2]. The peritoneal cavity is the most common location of ruptured LA, a serious surgical emergency [3].

Most LAs are observed in the right hepatic lobe and may be solitary or multiple. One of the following five methods of liver infection dissemination can be found in most LA cases: hematogenic, through the hepatic artery, portal vein, or umbilical vein in severe septic processes as in metastatic-pyemic LA or portal vascular thrombosis and occasionally as in omphalophlebitis; biliary, through the biliary tract in cases of cholecystitis or cholangitis, as well as from invasion by parasites (oriental cholangitis) or foreign bodies; by continuation, the spread of inflammatory processes to the liver from surrounding areas; post-traumatically, traumatic liver injuries or as a result of an intrahepatic hematoma; and postoperatively [1].

The primary cause of LA is appendicitis; however, with improved detection and treatment, the incidence of this condition has greatly declined. Currently, the main causes of PLAs include biliary tract disorders (biliary stones, strictures, malignancies, and congenital defects) [4]. The cause of a hepatic abscess may be difficult to determine, as the most common cause is cryptogenic. This is seen in 69% of all cases of LA, commonly presenting with comorbidities such as diabetes mellitus (60.9-68% of cases), coronary artery disease, chronic obstructive pulmonary disease, malignancy, or liver cirrhosis [5]. Blood tests are not diagnostic for LAs. However, leukocytosis and elevated alkaline phosphatase (ALP) levels are common findings [6]. Although there are no specific clinical criteria for differentiating between PLA and ALA, a differential diagnosis can be established using the following criteria: clinical suspicion of ALA increases with younger age, residency, or recent travel to locations with endemic amebiasis, diarrhea, and severe stomach discomfort [7]. LA rupture is a rare occurrence, with a prevalence of 5.4% [8]. Patients with LA often have non-specific constitutional symptoms when they first present, and their clinical presentation is atypical. The most frequent symptoms are fever and chills, followed by pain in the right upper quadrant and hepatic tenderness [9].

Compared with other LA etiologies, there is a higher incidence of abscess rupture in patients with Klebsiella pneumoniae PLA, notwithstanding the rarity of spontaneous LA ruptures. Persistent hyperglycemic dysregulation in patients with diabetes, large abscess size >5 cm, thinned wall, and gas-forming abscesses (pain limited to the right upper quadrant and hepatic tenderness) are suggested risk factors for spontaneous rupture in K. pneumoniae PLA [10].

When treatment is neglected and the diagnosis is delayed, a significant mortality rate of 75% has been seen [11]. Imaging remains essential for the diagnosis of ruptured LAs because it allows positive and topographic diagnoses. Surgery is always associated with medical treatment and remains the best option for cases of peritoneal cavity rupture. However, conservative percutaneous drainage with appropriate antibiotic therapy may be sufficient for localized rupture of the pleura, pericardium, or abdominal wall [12].

Any drainage strategy must include antibiotic medication because it plays a vital role. Given that E. coli, K. pneumoniae, and other Enterobacteriaceae have up to 30% resistance rates to fluoroquinolones, third-generation cephalosporins (ceftriaxone and cefotaxime) and piperacillin/tazobactam have solidified their leading positions in the treatment of PLA with antibiotics. Therefore, the first antibiotic regimen should consist of piperacillin, tazobactam, or third-generation cephalosporins and metronidazole. The latter has the advantage of partially covering enterococcal infections [10]. Based on available data, combined therapy involving an aminoglycoside and a beta-lactam antibiotic is recommended for individuals with severe infections caused by Klebsiella spp. and hypotension [13]. Carbapenems are recommended for treating extended-spectrum β-lactamase-producing K. pneumoniae and E. coli. Compared to other antibiotics, the use of carbapenems, particularly imipenem/cilastatin, is independently associated with lower mortality [14]. The increasing prevalence of K. pneumoniae strains resistant to carbapenems, such as those that produce the New Delhi metallo-beta-lactamase-1 or K. pneumoniae carbapenemases, is a concern because of the limited treatment options available for these hyperresistant strains, including tigecycline, colistin, and aminoglycosides. These strains are also associated with increased mortality [15]. Shorter antibiotic courses consisting of targeted intravenous therapy for two to three weeks and sequential oral therapy for one to two weeks have been linked to exceptionally low mortality rates of less than 5% in certain studies conducted in the USA. In cases of ALAs, metronidazole is an excellent agent, and most patients respond to therapy within 72-96 hours [16]. Studies have shown that percutaneous drainage should be considered the first choice, even in cases of multiple LAs, as it has demonstrated shorter hospitalization and lower costs than surgical drainage (laparotomy) [17].

## Materials and methods

Study design and patient selection

We retrospectively studied patients admitted to Safdarjung Hospital, New Delhi, India, between January 1, 2022, and December 31, 2023, reviewing the electronic medical records of patients with ruptured LAs. The inclusion criteria were as follows: confirmed LA (at least one space-occupying lesion in the liver) [1] through ultrasonography (USG) and patients aged >12 years. The exclusion criteria included outside-operated patients and those with missing data. This analysis included 115 patients with ruptured LAs.

Data collection and definition of the variables

Data on demographics (sex and age), duration and unit of hospitalization, clinical manifestations (pain, fever, jaundice, loose motions, obstipation, tenderness, peritonitis, melena), laboratory findings, and imaging at admission, comorbidities (diabetes mellitus, hypertension, and chronic liver diseases (CLDs), including hepatitis B and C), and alcohol consumption were collected. Routine blood examinations included a complete blood count, serum biochemical tests (including liver and renal function tests), coagulation profile, and serum albumin levels. Sepsis and septic shock were defined according to International Sepsis Definitions Conference criteria. Fever was defined as an ear temperature of >37.8 °C. Multiple LAs were defined as abscesses ≥3.

Treatment and outcomes

We collected data on the duration of inpatient intravenous antimicrobial therapy, surgical procedures performed, and pigtail (percutaneous USG-guided) drainage. Empirical antibiotic therapy included third-generation cephalosporins, piperacillin-tazobactam, and metronidazole. The evaluated treatment outcomes included antimicrobial therapy combined with percutaneous catheter drainage (PCD), surgical treatment, and intercostal chest tube drainage. The definition of PCD failure included death when an abscess drain was in place or when surgical treatment was required [15]. The need for surgical treatment was evaluated based on clinical expertise, an assessment of biochemical parameters, and hospital guidelines. Mortality included deaths that occurred in-hospital within 30 days of initial hospitalization. The cause of mortality was death as a direct consequence of PLA or its complications.

Statistical analyses

All data were analyzed using the IBM SPSS Statistics for Windows, Version 28 (Released 2021; IBM Corp., Armonk, New York, United States). Descriptive analysis was used to compare the differences in demographics, clinical manifestations, laboratory factors, and treatments (surgery and pigtail insertion). Continuous variables were presented as means (standard deviations (SDs)), while categorical data were presented as numbers (n) and percentages (%). Independent two-sample t-tests were used to analyze continuous variables. Categorical variables were compared using chi-square or Fisher’s exact tests. The relationships among demographic characteristics, clinical manifestations, laboratory factors, and outcomes were assessed using univariate analysis. Multivariate logistic regression models in the forward selection mode were applied to significant factors from the univariate analysis. All statistical tests were two-sided, and a p<0.05 was considered significant.

## Results

Demographics

**Figure 1:**
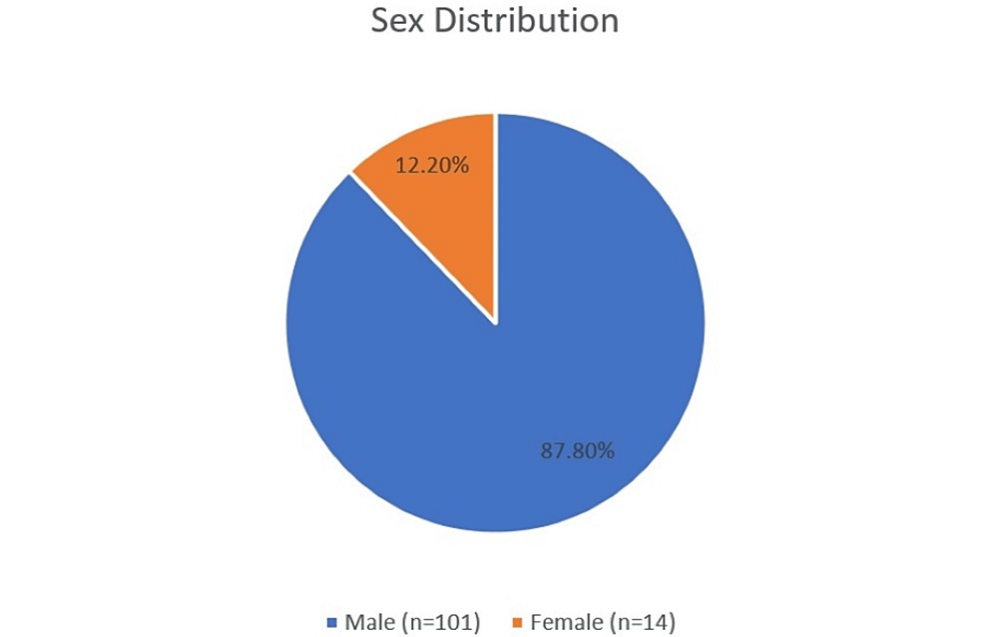
Sex distribution

**Figure 2:**
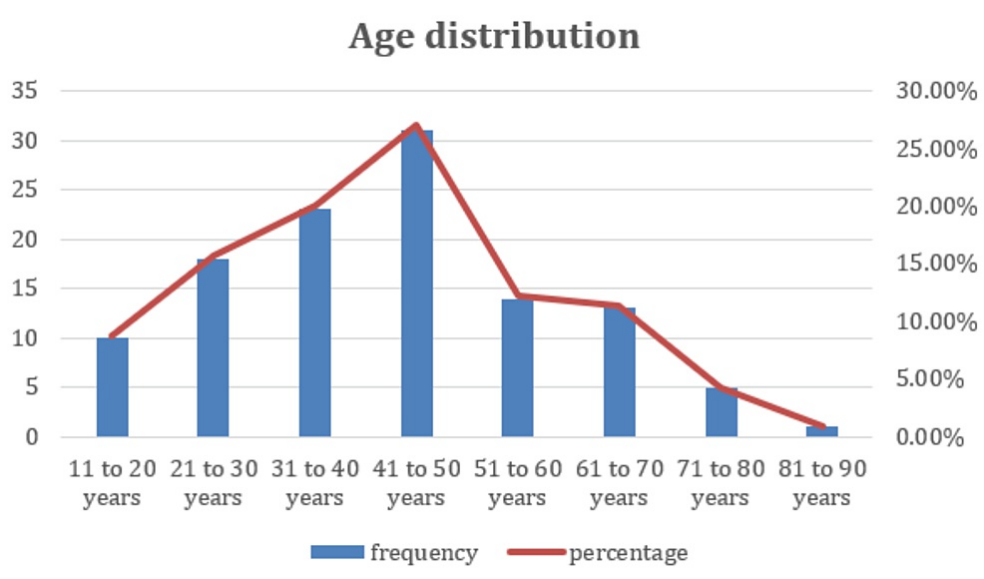
Age distribution (N = 115)

Of the 115 patients who were diagnosed with ruptured LAs in our study, 88% (n = 101) were male, and 12% (n = 14) were female (Figure 1). The most affected age group was 42-53 years (30 patients), with a median age of 45 years (Table 1, 2). The mean (±SD) age of the study population was 43.59 (±16.43) years (Figure 2).

**Table 1:** Age distribution of the study population (N = 115)

Age (years)	Frequency	Percentage
11–20	10	8.7%
21–30	18	15.7%
31–40	23	20.0%
41–50	31	27.0%
51–60	14	12.2%
61–70	13	11.3%
71–80	5	4.3%
81–90	1	0.9%

**Table 2:** Sex distribution of the study population (N = 115)

Sex	Percentage (N)
Male	87.8% (101)
Female	12.2% (14)

Clinical and laboratory findings

**Figure 3:**
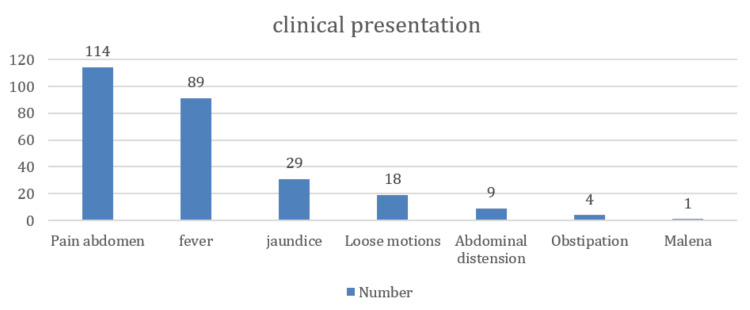
Distribution of chief complaints (N = 115)

Abdominal pain was the most common symptom (114 patients), and the average number of days of abdominal pain before the presentation was 14, with a median of 10 days (Table 3). Fever, jaundice, loose motions, and obstipation were present in 89 (77.3%), 29 (25%), 18 (15.6%), and four (3.4%) patients, respectively (Figure 3). Diabetes was present in 10 (8.6%) patients, 45 (39%) patients reported alcohol usage, and 26 (22.6%) patients had a history of smoking (Table 4). Eight patients were diagnosed with CLD, and one patient had a hepatitis C virus infection. Three patients had hypertension during treatment, and 12 (10.4%) patients had tuberculosis.

**Table 3:** Chief complaints of the study population (N = 115)

Clinical presentation	Number	Percentage
Abdominal pain	114	99.1%
Fever	89	77.4%
Jaundice	29	25.2%
Loose motions	18	15.7%
Obstipation	4	3.5%
Malena	1	0.8%

**Table 4:** Comorbidities of the study population (N = 115)

Comorbidities	Frequency	Percentage
Diabetes mellitus	10	8.7%
Hypertension	2	1.7%
Alcohol consumption	45	39.1%
Smoking	26	22.6%
Chronic liver disease	8	7.0%
Undernutrition	2	1.7%
Tuberculosis	12	10.4%

Right upper quadrant tenderness was the most common clinical finding present in 106 (92.1%) of the 115 patients, and three patients presented with shock. Generalized guarding and peritonitis were present in 30.4% (n = 35) of the patients.

Seven patients had severe anemia. Anemia (hemoglobin (HB) <10 g/dL), leukocytosis, and raised serum creatinine were present in 60 (52.1%), 77 (67%), and 32 (27.8%) patients, respectively. The international normalized ratio (INR) was atypical in 54 (46.9%) patients. One hundred (87%) patients had elevated ALP levels. Pleural effusion was observed in 62 (53.9%) patients (Table 5).

**Table 5:** Biochemical parameters of the study population (N = 115) ALP: alkaline phosphatase; TLC: total leukocyte count; SD: standard deviation; PT/INR: prothrombin time/international normalized ratio

Parameters	Summary statistics
Hemoglobin, Mean±(SD)	10.43±(2.09)
TLC, Mean±(SD)	21.3±(27.4)
Creatinine, Mean±(SD)	1.17±(1.13)
PT/INR, Mean±(SD)	1.5±(0.4)
Bilirubin, Mean±(SD)	1.9±(2.8)
Albumin, Mean±(SD)	1.8±(0.94)
ALP, Mean±(SD)	327.7±(314)

Characteristics of ruptured LA and collection

**Figure 4:**
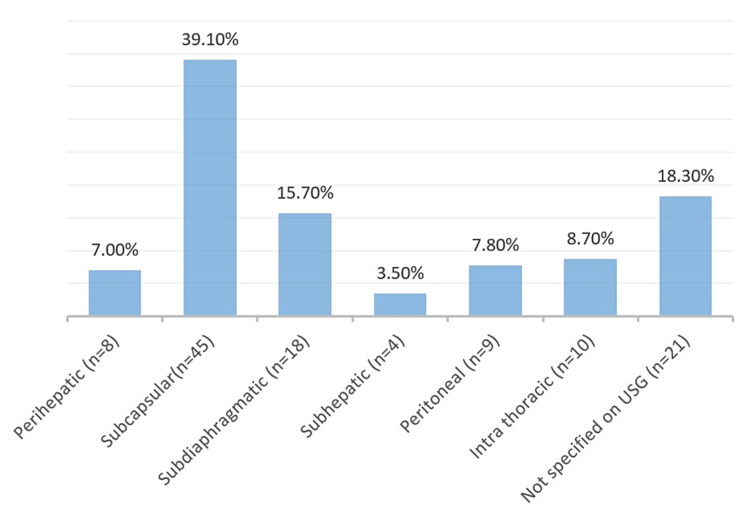
Distribution of ruptured collection

On the USG, 79 (68.6%) patients had a single cavity. The right and left lobes were isolated in 88 (76%) and 11 (9.5%) patients, respectively (Table 6, 7). Combined right and left lobe involvement was observed in 12 (10%) patients. Subcapsular collection with rupture was present in 45 (39.1%) patients; other locations were perihepatic in eight patients, subdiaphragmatic in 18 patients, subhepatic in four patients, peritoneal in nine patients, and intrathoracic in 10 patients, with 8.6% having a pleural connection (Table 8, Figure 4).

**Table 6:** Liver lobe distribution

Site	Frequency	Percentage
Right	88	76%
Left	11	9.5%
Both right and left	12	10%

**Table 7:** Number of abscess cavity

Liver abscess	Frequency	Percentage
Single	84	73%
Multiple	31	26.90%

**Table 8:** Ultrasound findings in the location of ruptured liver abscess USG: ultrasonography

Location of collection	Frequency	Percentage
Perihepatic	8	7.0%
Subcapsular	45	39.1%
Subdiaphragmatic	18	15.7%
Subhepatic	4	3.5%
Peritoneal	9	7.8%
Intra thoracic	10	8.7%
Not specified on USG	21	18.3%

Isolated segmental distribution of LA

Segments 5, 6, 7, and 8 were involved in six, eight, 12, and 10 cases, respectively; however, in 33 cases with multiple-segment involvement, segments 5, 6, and 7 were the most common locations (Table 9). The average volume of LA collection recorded on USG was 292 cc, with a maximum of 1,500 cc recorded in one patient. Moreover, 103 (89%) patients were diagnosed with ruptured LAs, as rent was visible along the cavity wall.

**Table 9:** Segmental distribution of ruptured liver abscess on abdominal ultrasound

Right lobe liver abscess		Frequency	(%)
Segment	Segment not identified	13	11.3
	Segment 5	6	5.2
	Segment 6	8	7
	Segment 7	12	10.4
	Segment 8	10	8.7
	Segment 9	1	0.9
	Segment 10	19	16.5
	Segment 5+6	3	2.6
	Segment 5+7	2	1.7
	Segment 6+7	18	15.7
	Segment 5+8	1	0.9
	Segment 7+8	12	10.4
	Segments 6, 5, and 7	1	0.9
	Segments 7, 8, and 5	2	1.7
	Segments 6, 7, and 8	2	1.7
	Segments 5, 6+7, and 8	1	0.9
	Caudate lobe	1	0.9
	Segment 4+8	1	0.9
	Segments 5 and 7+caudate	1	0.9
	Segment 8+6	1	0.9
	Total	115	100

Management, treatment, and outcomes

**Figure 5:**
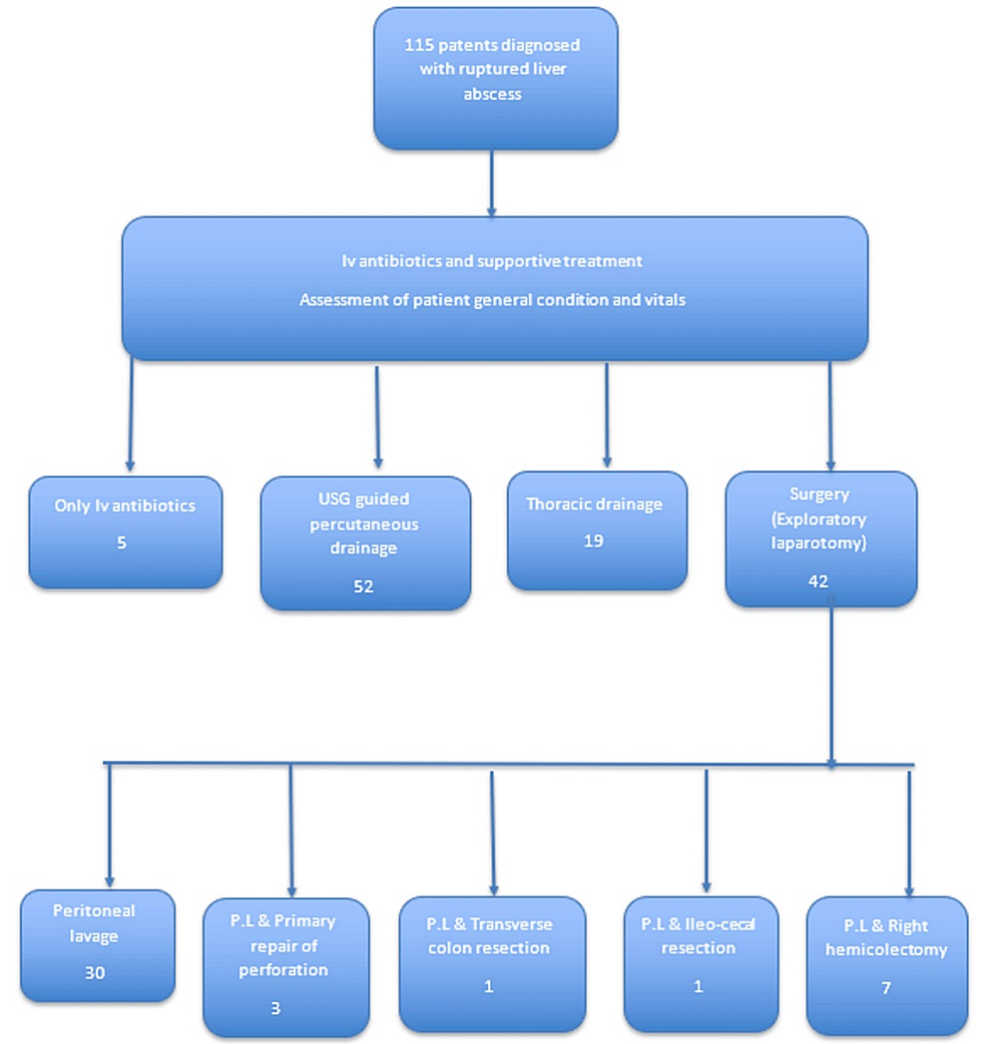
Distribution of management options in patients with ruptured liver abscess PL: peritoneal lavage; iv: intravenous

Five (4.3%) patients were discharged after receiving intravenous antibiotic treatment (Figure 5). Fifty-two (45.2%) patients underwent USG-guided percutaneous drainage, and one patient underwent USG-guided aspiration. Forty-two (36.5%) patients underwent surgery (exploratory laparotomy) (Table 10). Sixteen (13.9%) patients had large bowel perforations (Table 11). Nine patients had cecal perforation, three patients had cecal and ascending colon perforation, and one patient had hepatic flexure perforation. Three patients had isolated transverse colonic perforations (Table 12). Two patients underwent laparotomy after unsuccessful percutaneous drainage. An intraoperative pleural rupture was observed in one patient. Nineteen patients required thoracic drainage (intercostal drainage (ICD) tube), seven required percutaneous drainage and ICD, and three required laparotomy and ICD. Twenty-three (20%) patients were reported dead, including 17 patients who underwent laparotomy and nine patients who had large bowel involvement/perforation (39.1% associated with overall mortality, 52.9% associated with mortality in laparotomy). One patient who underwent percutaneous drainage and a right ICD tube placement was admitted to the intensive care unit. Four patients were declared dead before any intervention could be performed, representing 17.3% of the total 23 mortality cases, which is 3.4% of the total 115 patients included in this study, which signifies the severity of the disease at presentation. In one patient, the ICD tube was secured, given respiratory distress.

**Table 10:** Procedure performed in the study population (N = 115)

Treatment	Frequency	Percentage
Medical alone	5	4.3%
Guided aspiration	1	0.8%
Total thoracic drainage done	19	16.5%
Percutaneous drainage	52	45.2%
Surgery	42	36.5%
Fallout from study	1	0.8%

**Table 11:** Large bowel perforation in ruptured liver abscess

Perforation	Frequency	Percentage
Cecum	9	7.8%
Cecum and ascending colon	3	2.6%
Transverse colon	3	2.6%
Hepatic flexure	1	0.9%
No	99	86.1%

**Table 12:** Surgical procedure performed

Surgical procedure	Frequency	Percentage
Drainage of ruptured liver abscess	30	26.%
Right hemicolectomy with double barrel ileo-transverse stoma	7	6%
Primary repair of cecal/colon perforation with end ileostomy	3	2.6%
Transverse colon resection with end transverse colostomy	1	0.8%
Ileocecal resection with end ileostomy	1	0.8%
Total	42	36.5%

Predictors of surgical drainage

Surgery was performed in 42 patients, and the associations between surgery and clinical, biochemical, and radiological parameters were evaluated.

Loose motion (p = 0.018), alcohol consumption (p = 0.003), smoking (p = 0.037), HB level (p = 0.002), creatinine level (p = 0.00), ALP (p = 0.009), albumin level (p = 0.004), volume (p = 0.027), and diastolic blood pressure (p = 0.04) were significantly associated (Table 13).

**Table 13:** Clinical, biochemical and radiological correlation with cases of open drainage (surgical management) CXR: chest X-ray; HB: hemoglobin; TLC: total leukocyte count; S.bil: S.Bilirubin; ALP: alkaline phosphatase; DM: diabetes mellitus; ml: mililiter; dl-deciliter; g: gram; mg: miligram; IU: international units; mm: millimeter; r: correlation coefficientl INR: international normalized ratio; SBP: systolic blood pressure; DBP: diastolic blood pressure

Characteristics	Output	P-value	r value
Number	42		
Age ±SD (years)	42±18	0.249	
Sex (male)	38		-0.061
Sex (female)	4		
Pulse (/min)	101±18	0.025	
SBP (mmHg)	115±18	0.280	
DBP (mmHg)	70±10	0.070	
Clinical parameters	%(n)		
Tenderness (106)	37.7% (n=40)	0.353	0.087
Peritonitis (66)	78% (n=33)	0.000	0.108
Loose motions (18)	61.1% (n=11)	0.018	0.220
Jaundice (29)	48.3% (n=14)	0.12	0.142
Fever (89)	39% (n=35)	0.240	0.108
Obstipation (4)	75% (n=3)	0.137	0.152
Biochemical parameters	Mean±SD		
Hemoglobin	11±2	0.002	
TLC	29±43	0.117	
Platelets	11±23	0.183	
Creatinine	1.18±0.8	0.00	
INR	1.4±0.4	0.520	
Serum bilirubin	1.8±2.6	0.470	
Albumin	1.6±0.6	0.004	
ALP	273±150	0.009	
Comorbidities	%(n)		
Chronic liver disease (8)	50% (n=4)	0.412	0.077
Diabetes mellitus (10)	20% (n=2)	0.256	-0.106
Alcohol consumption (45)	53% (n=24)	0.003	0.280
Smoking (26)	53.8% (n=14)	0.037	0.194
Pus (mL)	931±638	0.027	

Predictors of mortality

A total of 23 (20%) deaths were recorded: loose motion (p = 0.029), jaundice (p = 0.00), pleural effusion on chest X-ray (CXR) (p = 0.026), perforation (p = 0.001), CLD (p = 0.047), peritonitis (p = 0.045), age (p = 0.006), pulse (p = 0.0029), systolic blood pressure (p = 0.002), diastolic blood pressure (p = 0.00), total leukocyte count (p = 0.002), creatinine (p = 0.001), INR (p = 0.009), and albumin (p = 0.028) (Table 14, 15). A significant association was found regarding the location of the ruptured abscess (p = 0.024): 30.4% (n = 7) had subcapsular rupture (n = 45), and 26.1% (n = 6) had peritoneal rupture (n = 9). Patients who underwent surgery had a high risk of mortality of 73.9% (n = 17). Cecal perforation accounted for 66.7% (n = 9) of all documented cases of perforation.

**Table 14:** Outcome in the study population (N = 115) LAMA: leave against medical advice

Outcome	Frequency	Percentage
Death	23	20.00%
Discharge	90	78.30%
LAMA	2	1.70%

**Table 15:** Association of clinical, biochemical parameters with mortality in cases of ruptured liver abscess The data are presented as percentage (%), total number (n), and mean±SD, and p<0.05 indicated significance. CXR: chest X-ray; TLC: total leukocyte count; ALP: alkaline phosphatase; SD: standard deviation; INR: international normalized ratio

Characteristics	Mortality	P-value
Number	23	
Age±SD (years)	51±16	0.006
Sex (male)	20 (87%)	0.038
Sex (female)	3 (13%)	
Clinical parameters	%(n)	
Tenderness (106)	19.8% (n=21)	0.862
Peritonitis	40.9% (n=9)	0.045
Loose motions (18)	38.9% (n=7)	0.029
Jaundice (29)	44.8% (n=13)	0.000
Fever (89)	21% (n=19)	0.540
Obstipation (4)	50% (n=2)	0.178
Biochemical parameters	Mean±SD	
Hemoglobin	10±2	0.381
TLC	35±57	0.002
Platelets	10.5±54	0.353
Creatinine	2.0±1.6	0.001
INR	1.7±0.6	0.009
Serum bilirubin	2.7±2.8	0.077
Albumin	1.5±0.6	0.028
ALP	340±170	0.417
Comorbidities	%(n)	
Chronic liver disease (8)	50% (n=4)	0.028
Diabetes mellitus (10)	20 % (n=2)	1.0
Alcohol consumption (45)	26.5% (n=12)	0.152
Smoking (26)	11.5% (n=3)	0.220
Pus (mL)	1,095±661	0.129
Pleural effusion (CXR) (62)	25.8% (n=16)	0.092

Predictors of perforation

Small bowel perforation was observed in 16 patients, and jaundice (0.036), loose motion (0.013), alcohol consumption (0.034), CLD (0.044), peritonitis (p = 0.006), fever (p = 0.038), and tenderness (p = 0.004) were found to be significant (Tables 16, 17).

**Table 16:** Biochemical variables with perforation in ruptured liver abscess The data are presented as percentage (%), total number (n), and mean±SD, and p<0.05 was considered significant. TLC: total leukocyte count; ALP: alkaline phosphatase; SD: standard deviation; DBP: diastolic blood pressure; SBP: systolic blood pressure; PT/INR: prothrombin time/international normalized ratio

Parameter	Case	N	Mean	SD	P-value
Age	With perforation	16	48.56	16.597	0.097
	No perforation	99	42.79	16.343	
Pulse (/min)	With perforation	16	100.19	16.477	0.253
	No perforation	99	96.95	18.280	
SBP (mmHg)	With perforation	16	115.63	17.193	0.407
	No perforation	99	116.71	17.053	
DBP (mmHg)	With perforation	16	73.50	13.525	0.416
	No perforation	99	72.86	10.733	
Hemoglobin (g/dL)	With perforation	16	11.425	1.9077	0.020
	No perforation	99	10.268	2.0824	
TLC (*10^3/mm^3)	With perforation	16	37.588	69.7691	0.148
	No perforation	99	18.689	9.1390	
Platelet (lakh/mm^3)	With perforation	16	20.407500	32.8645003	0.069
	No perforation	99	7.334122	15.9605138	
Creatinine (mg/dL)	With perforation	16	1.4731	1.15854	0.128
	No perforation	99	1.1232	1.13208	
PT/INR	With perforation	16	1.5494	0.47901	0.425
	No perforation	99	1.5259	0.45880	
Bilirubin (mg/dL)	With perforation	16	1.4463	1.23314	0.211
	No perforation	99	2.0566	2.97583	
ALP (IU/L)	With perforation	16	348.81	171.693	0.387
	No perforation	99	324.30	331.982	
Albumin (g/dL)	With perforation	16	1.525	0.6434	0.053
	No perforation	99	1.938	0.9800	
Volume (mL)	With perforation	16	292.50	260.283	0.327
	No perforation	94	323.60	255.021	

**Table 17:** Association of clinical parameters with perforation The data are presented as percentage (%), total number (n), and mean±SD; p<0.05 was considered significant. CXR: chest X-ray

Characteristics	% (n)	P-value
Number (115)	13.9 (16)	
Sex (male) 101	87.5 (14)	0.966
Sex (female) 14	12.5 (2)	
Clinical parameters	%(n)	
Loose motions (18)	15.5 (5)	0.013
Jaundice (29)	25.2 (9)	0.036
Fever (89)	30.4 (6)	0.038
Obstipation(4)	0	0.955
Tenderness (106)	92 (14)	0.004
Peritonitis (25)	57 (12)	0.006
Comorbidity	%( n)	
Chronic liver disease (8)	7 (2)	0.044
Diabetes mellitus (10)	0	0.778
Alcohol consumption (45)	39.1 (9)	0.034
Smoking (26)	22.6 (4)	0.342
Pus (mL)	1131±713	0.089
Pleural effusion (CXR) (62)	53.9 (8)	0.332

A total of 23 mortalities were recorded, of which 11 (68.8% of perforation cases) were found to involve perforation, and five patients with perforation survived with prompt management (p = 0.000).

Ruptured abscess status on USG

A ruptured LA, other than systolic blood pressure, was reported on USG in 105 patients, and no significant association was found between demographic, clinical, and biochemical parameters (Table 18).

**Table 18:** Correlation of ruptured status on USG abdomen with clinical and biochemical parameters The data are presented as percentage (%), total number (n), and mean±SD, and p<0.05 was considered significant. TLC: total leukocyte count; ALP: alkaline phosphatase; SD: standard deviation; INR: international normalized ratio; DBP: diastolic blood pressure; SBP: systolic blood pressure; CXR: chest X-ray

Characteristics	Rupture liver abscess	P-value
Number	105	
Age±SD (years)	43±16	0.459
Sex (male)	88.6% (93)	0.428
Sex (female)	11.4% (12)	
Clinical parameters		
Tenderness (106)	91.4% (96)	0.335
Peritonitis (25)	32% (24)	0.122
Loose motions (18)	17.1% (18)	0.154
Jaundice (29)	24.8% (26)	0.716
Fever (89)	76.2% (80)	0.312
Obstipation (4)	2.9% (3)	0.229
Pulse (/min)	98±18	0.071
SBP (mmHg)	115±16	0.029
Biochemical parameters	Mean±SD	
Hemoglobin	10±2	0.101
TLC	21±28	0.265
Platelets	9.4±20.2	0.276
Creatinine	1.19±1.17	0.289
INR	1.5±0.47	379
Serum bilirubin	1.8±2.7	0.115
Albumin	1.9±0.9	0.243
ALP	328±323	0.475
Comorbidity	%(n)	
Chronic liver disease (8)	6.7% (7)	0.692
Diabetes mellitus (10)	8.6% (9)	0.878
Alcohol consumption (45)	41% (43)	0.195
Smoking (26)	21.9% (23)	0.559
Pus (mL)	950+/-663	0.471
Pleural effusion (CXR) (62)	88.7% (55)	0.278

## Discussion

Ruptured LA is an uncommon complication of LA, and this current study showed a 20% mortality rate for cases that were not diagnosed promptly and treated appropriately, which was less than the 43% reported by Chou et al. [8]. The 20% mortality rate reported in patients with ruptured LAs in this study was higher than the rate reported in a study by Ibarra-Pérez [18]. The hospital where this study was conducted is the major referral center that receives patients from all over India, and some of these referrals have a late presentation of the disease, which may account for this difference (high mortality rate). The natural course of an LA is rupture, which can be fatal if left untreated.

Of the 115 patients in this study, 88% were male. Abdominal pain was the most common complication in our study, with a median presentation time of 14 days, as reported by Rajak et al. [19]. Common comorbidities observed in patients with LAs include diabetes mellitus, hypertension, malignant tumors, biliary stones, a history of abdominal surgery, liver cirrhosis, and alcoholism [20]. In our study, 40% of the population consumed alcohol. The following mechanisms have been proposed for the ALA predisposition to alcohol: alcohol suppression of Kupffer cell function, which plays an essential role in clearing the ameba; hepatocyte damage by alcohol; hepatic accumulation of iron; alcohol-facilitated invasive capacity of Entamoeba histolytica (EH); nutritional deficiency-induced lowered body resistance and suppression of liver function in patients who consume alcohol; alcohol-associated depression of immune activity; and facilitation of EH entry into the blood by dysbiosis of intestinal bacteria and alcohol-induced intestinal hyperpermeability [21].

Abdominal pain was the most common complaint, followed by fever, jaundice, and loose motions, which matched the results of other studies where fever was the most common presentation and was seen in 91% of patients, followed by abdominal pain in 86%, loss of appetite in 43%, jaundice in 36%, nausea and vomiting in 20%, and a few other symptoms, including cough, expectoration, loose motions, and abdominal mass. These findings are in line with those of Ochsner et al., who reported that 94% of their study population suffered from fever, 92% experienced abdominal pain, and 33% had nausea and vomiting [22]. Greenstein et al. observed that 95%, 84%, 42%, 39%, and 24% of patients with LAs had a fever, abdominal pain, abdominal tenderness, hepatomegaly, and jaundice, respectively [23]. In our study, LAs were confined to the right lobe in 76% of the cases, which is in agreement with the study conducted by Sharma et al. [24].

Leukocytosis, elevated ALP levels, anemia, and an atypical INR are common laboratory findings in ruptured abscesses. Pleural effusion was observed in more than 50% of the cases. A ruptured abscess requires drainage; 45.2% of the patients responded to percutaneous drainage, and 36.5% of the patients required surgical management. Only two patients had failed PCD; they underwent surgical drainage. In our setting, if a patient is in shock with features of peritonitis, a USG diagnosis of a ruptured LA is indicated for an emergency exploratory laparotomy. Patients with leukocytosis, stable vitals (only tachycardia and fever), and right upper abdominal tenderness underwent percutaneous drainage, with a daily assessment of drain output and the development of sepsis. Patients with only abdominal pain, a USG diagnosis of ruptured LA, and a near-normal biochemical profile are candidates for USG-guided pigtail/percutaneous drainage.

A significant association was found between the presenting complaints and clinical and biochemical parameters for the patient who underwent surgery, including loose motion (p = 0.018), alcohol consumption (p = 0.003), smoking (p = 0.037), HB (p = 0.002), creatinine (p = 0.00), ALP (p = 0.009), albumin (p = 0.004), volume (p = 0.027), and diastolic blood pressure (p = 0.04).

A significant association was found in patients who were reported to have died. Twenty-three deaths were recorded (20%), and loose motion (p = 0.029), jaundice (p = 0.00), pleural effusion on CXR (p = 0.026), perforation (p = 0.001), CLD (p = 0.047), peritonitis (p = 0.045), age (p = 0.006), pulse (p = 0.0029), systolic blood pressure (p = 0.002), diastolic blood pressure (p = 0.002), total leukocyte count (p = 0.002), creatinine (p = 0.001), INR (p = 0.009), and serum albumin (p = 0.028) showed statistical significance.

We recognize that the retrospective nature of our study serves as a limitation. Our information was restricted to what was documented in the medical files. Nonetheless, no notable distinction existed in the absent assessments at admission between the groups with and without case fatalities. The potential impact of the non-random effect of missing data on the research findings was reduced to a minimum. In our study, serum creatinine was a significant prognostic factor for mortality, which is consistent with previous findings [25]. In a few studies, age, blood pressure, white blood cell count, hematocrit, serum creatinine levels, and underlying medical conditions (malignancy, liver cirrhosis, and uremia) were independent predictors of mortality, as shown in our study. As microbiological examinations were not performed in our study, the risk of K. pneumoniae, gas-forming abscesses, and associated complications (such as thrombocytopenia and endophthalmitis), which can affect mortality, was not assessed.

The novelty of this study lies in its identification of a significant independent predictor of bowel perforation in patients with jaundice (p = 0.036), loose motion (p = 0.013), alcohol consumption (p = 0.034), CLD (p = 0.044), peritonitis (p = 0.006), fever (p = 0.038), and tenderness (p = 0.004).

In a study published in India in the 1970s, Monga et al. described two patterns of rupture associated with amebic peritonitis [26]. They were convinced that rupture cases could be managed conservatively, whereas free rupture (intraperitoneal spread) required open drainage. Subsequently, a new study reportedly showed that the two distinct patterns affected the outcome and that a conservative approach was sufficient to manage localized intraperitoneal fluid collection; however, 50% of cases with generalized peritonitis in their study required surgery [27].

In our study, 45.2% (n = 52) of the patients underwent percutaneous drainage, and only two reported treatment failure, which was in line with the study conducted by Sohn et al. [28]. This study also considered the outcomes of patients who underwent percutaneous drainage, as more than 50% were treated with the same modality, with a decreased total duration of hospital stay. At our institute, PCD is the treatment of choice for uncomplicated ruptured LAs.

A limitation of this study is its retrospective nature and single-center design, which can only help to create a hypothesis regarding the factors significantly associated with perforation and mortality and the patients that might require open surgical drainage. Therefore, further prospective trials are required to confirm this hypothesis. Nevertheless, our study has some strengths. First, it adds to the factors already defined in the literature and provides an in-depth analysis of the factors associated with surgical drainage and bowel perforation linked to ruptured LAs.

Depending on the type of rupture, these authors attempted to make recommendations regarding the appropriate surgery for ruptured ALA. However, the mortality associated with surgery in the management of amebic peritonitis remained high and was reported to be up to 50% in a study by Priyadarshi et al. All free rupture cases were treated with catheter drainage; however, they required multiple catheters and longer hospital stays, and they showed that incomplete drainage in 50% of the patients was helpful in relieving peritonitis. Thus, complex septation does not preclude PCD as a therapeutic option for ALA rupture in generalized peritonitis. An intercostal chest tube may be required if empyema is suspected because of pleural communication of a ruptured abscess or massive pleural effusion causing respiratory distress.

## Conclusions

A ruptured LA is a surgical emergency with varied clinical presentations, from only abdominal pain to features of perforation, peritonitis, and shock. Cryptogenic LAs are the most common. Males were most affected, usually involving the middle-aged group (the median age in our study was 45 years). Alcohol consumption is a major risk factor for LAs. Early recognition of clinical symptoms and prompt investigation and treatment of sick patients were prognostic indicators. Tenderness, peritonitis, loose motion, increasing age, increased creatinine, leukocytosis, low albumin levels, and a history of CLD were independent predictors of mortality. Prompt treatment should be initiated, and adequate resuscitation should be performed to prevent and decrease LA-associated mortality. Jaundice, history of loose motion, alcohol consumption, fever, and CLD were significantly associated with perforation; caution should be taken in these cases, and prompt surgical intervention should be performed to correct the underlying pathology. USG-guided percutaneous drainage is the preferred treatment for stable patients without peritoneal features. Surgical management and exploratory laparotomy are life-saving procedures performed on an emergency basis to reduce the source and ensure adequate abscess drainage. Good knowledge of the symptoms, signs, and biochemical profiles can guide early treatment.
